# Bright Blue, Green, and Red Luminescence from Dye-Sensitized Core@Shell Upconversion Nanophosphors under 800 nm Near-Infrared Light

**DOI:** 10.3390/ma13235338

**Published:** 2020-11-25

**Authors:** A-Ra Hong, Joon Soo Han, Gumin Kang, Hyungduk Ko, Ho Seong Jang

**Affiliations:** 1 Materials Architecturing Research Center, Korea Institute of Science and Technology, 5, Hwarang-ro 14-gil, Seongbuk-gu, Seoul 02792, Korea; 024446@kist.re.kr (A.-R.H.); jshan@kist.re.kr (J.S.H.); 2 Nanophotonics Research Center, Korea Institute of Science and Technology, 5, Hwarang-ro 14-gil, Seongbuk-gu, Seoul 02792, Korea; guminkang@kist.re.kr (G.K.); kohd94@kist.re.kr (H.K.)

**Keywords:** upconversion nanophosphors, core@shell, IR-808 dye, dye-sensitized upconversion nanophosphors

## Abstract

In this study, Li-based blue- and green-emitting core@shell (C@S) upconversion nanophosphors (UCNPs) and NaGdF_4_-based red-emitting C@S UCNPs were synthesized, and IR-808 dyes were conjugated with the C@S UCNPs to enhance upconversion (UC) luminescence. The surface of the as-synthesized C@S UCNPs, which was originally capped with oleic acid, was modified with BF_4_^−^ to conjugate the IR-808 dye having a carboxyl functional group to the surface of the UCNPs. After the conjugation with IR-808 dyes, absorbance of the UCNPs was significantly increased. As a result, dye-sensitized blue (B)-, green (G)-, and red (R)-emitting UCNPs exhibited 87-fold, 10.8-fold, and 110-fold enhanced UC luminescence compared with B-, G-, and R-emitting Nd^3+^-doped C@S UCNPs under 800 nm near-infrared (NIR) light excitation, respectively. Consequently, dye-sensitized UCNPs exhibiting strong UC luminescence under 800 nm NIR light excitation have high applicability in a variety of biological applications.

## 1. Introduction

Lanthanide-doped upconversion nanophosphors (UCNPs) have been widely used in biological applications owing to their unique features such as large anti-Stokes shift luminescence under invisible near-infrared (NIR) light and non-cytotoxicity [1,2,3,4,5,6,7,8,9]. Tang’s group reported the results of in vivo upconversion (UC) luminescence/magnetic resonance imaging using NaYF_4_:Yb,Er@NaGdF_4_@PEG-CD326 micelles under a 980 nm NIR laser [10]. Our group synthesized Li(Gd,Y)F_4_:Yb,Er@LiGdF_4_ core@shell (C@S) UCNPs and modified the surface of the C@S UCNPs with poly(acrylic acid) for their dispersion in water [9]. In this study, in vitro cell imaging and in vivo imaging were performed with 980 nm NIR light. In general, the Yb^3+^ ions, which are used as sensitizers, absorb NIR light at 980 nm. However, under irradiation with 980 nm NIR light for a long time, an overheating problem, that is, the increase of the temperature of the biomolecules, can cause cell death [11]. To solve this problem, many researchers have studied 800 nm-excitable C@S or core@multi-shell UCNPs in which the shell is doped with Nd^3+^ ions because Nd^3+^ ions have a high absorption cross-section at around 800 nm [1,12,13,14,15,16,17,18,19]. Almutairi’s group synthesized a high concentration of Nd^3+^-doped C@S UCNPs that showed blue and green UC luminescence (UCL) under 800 nm NIR light [16]. In addition, Hirsch’s group reported Yb^3+^/Er^3+^-doped core and Yb^3+^/Nd^3+^-doped C@S UCNPs, and the synthesized C@S UCNPs showed green emission under 980 nm and 800 nm excitation, respectively [18]. Previously, our group reported the sub-20 nm-sized red-emitting NaGdF_4_:Yb,Ho,Ce@NaYF_4_:Nd,Yb@NaGdF_4_ core@double-shell UCNPs and we showed in vivo UCL imaging and magnetic resonance imaging results utilizing the core@double-shell UCNPs [1]. Recently, NIR dye-sensitized UCNPs have been studied to significantly increase the absorption at around the 800 nm region and hugely enhance the emission intensity [20,21,22,23,24,25]. Wu’s group synthesized C@S UCNPs and showed that the UCL from the C@S UCNPs was largely enhanced under 820 nm NIR excitation by the conjugation of IR-820 dye to the C@S UCNPs [21]. Prasad’s group reported that the energy absorbed by the organic dye is efficiently transferred to Tm^3+^ ions doped in the core UCNPs through Nd^3+^ ions doped in the shell. Through Nd^3+^-mediated efficient energy transfer from the organic dye to the UCNP core, Tm^3+^ luminescence was significantly enhanced [22]. Jiang’s group synthesized IR-806 sensitized NaYF_4_:Yb,Er@NaYF_4_:Yb,Nd C@S UCNPs and they reported temperature-sensing capability of the IR-806 dye-sensitized C@S UCNPs [23]. In contrast, Kong’s group showed that IR-806 dye-conjugated NaYF_4_:Yb,Er@NaYbF_4_:Nd(20%) C@S UCNPs have potential for an anti-counterfeiting application [24]. In 2017, Lin’s group exhibited that mesoporous silica coated IR-808-sensitized green-emitting UCNPs and applied the UCNPs as agents for photodynamic therapy [25]. As described above, NIR dye-sensitization is beneficial to the enhancement of the luminescence from UCNPs. However, previous studies on dye-sensitized UCNPs were based on NaYF_4_-based blue- and green-emitting UCNPs, and non-NaYF_4_-based dye-sensitized UCNPs have not yet been studied [20,21,22,23,24,25]. In addition, to the best of our knowledge, no study has been reported on dye-sensitized red-emitting UCNPs. These results encouraged us to investigate non-NaYF_4_-based dye-sensitized UCNPs and red-emitting dye-sensitized UCNPs.

In this study, we synthesized Li(Gd,Y)F_4_-based blue- and green-emitting C@S UCNPs and NaGdF_4_-based red-emitting C@S UCNPs. Subsequently, the IR-808 dye was conjugated to the synthesized C@S UCNPs to implement bright blue, green, and red UCL since bright UCL from UCNPs under 800 nm excitation will be more beneficial for bio-imaging applications due to the minimized heating effect on the cells and tissues [11]. The UCL properties of the IR-808 dye-sensitized C@S UCNPs were investigated and their UCL intensities were significantly enhanced compared with the C@S counterparts.

## 2. Materials and Methods 

For the syntheses of core and C@S UCNPs, GdCl_3_·6H_2_O (99%), YCl_3_·6H_2_O (99.99%), YbCl_3_·6H_2_O (99.9%), TmCl_3_·6H_2_O (99.99%), ErCl_3_·6H_2_O (99.99%), HoCl_3_·6H_2_O (99.9%), CeCl_3_·7H_2_O (99.999%), NdCl_3_·6H_2_O (99.9%), LiOH·H_2_O (99.995%), NaOH (99.99%), NH_4_F (≥99.99%), 1-octadecene (ODE, 90% technical grade), and oleic acid (OA, 90% technical grade) were purchased from Sigma-Aldrich (St.Louis, MO, USA). Sodium oleate was purchased from TCI (Tokyo, Japan). For the synthesis of the IR-808 dye, IR-783 dye (90%) and 4-mercaptobenzoic acid (99%) were obtained from Sigma-Aldrich (St.Louis, MO, USA).

First, the IR-808 dye was synthesized using IR-783 dye and 4-mercaptobenzoic acid, as reported by Parasad’s group [22].

The blue-emitting Li(Gd,Y)F_4_:Yb,Tm core UCNPs were synthesized using rare-earth oleate (RE-oleate) precursors that were prepared by adapting a previously reported method presented by Hyeon’s group [26]. To synthesize RE-oleate precursors, GdCl_3_·6H_2_O (0.25 mmol), YCl_3_·6H_2_O (0.49 mmol), YbCl_3_·6H_2_O (0.25 mmol), TmCl_3_·6H_2_O (0.01 mmol), and sodium oleate (3.1 mmol) were dissolved into deionized water (DIW, 3 mL), ethanol (3.5 mL), and hexane (7 mL), and the solution was reacted at 70 °C for 4 h. The RE-oleate precursors were mixed with OA (10.5 mL) and ODE (10.5 mL), and the temperature of the mixture was increased to 150 °C for 40 min. The reaction solution was cooled down to 50 °C and then 10 mL of the methanol (MeOH) solution, which contained LiOH·H_2_O (2.5 mmol) and NH_4_F (4 mmol), were injected into the reaction solution. After the MeOH was removed, the reaction solution was reacted at 320 °C for 90 min under argon (Ar) atmosphere. The synthesized core UCNPs were dispersed in 10 mL of non-polar solvents such as chloroform and hexane after washing with ethanol.

To synthesize Li(Gd,Y)F_4_:Yb,Tm@LiYF_4_:Nd,Yb C@S UCNPs, the RECl_3_·6H_2_O (RE = Y (0.45 mmol), Nd (0.5 mmol), Yb (0.05 mmol)) precursors were mixed with OA (10.5 mL) and ODE (10.5 mL), and the mixture was reacted at 150 °C for 40 min. The mixed solution was cooled to 60 °C and the Li(Gd,Y)F_4_:Yb,Tm core UCNP solution (10 mL) was added to the mixed solution. Subsequently, the LiOH·H_2_O (2.5 mmol) and NH_4_F (4 mmol) dissolved MeOH solution (10 mL) was added followed by heat treatment at 320 °C for 60 min under Ar atmosphere. The C@S UCNPs were dispersed in 10 mL of chloroform after washing with MeOH, ethanol, and hexane.

To synthesize Li(Gd,Y)F_4_:Yb,Er core UCNPs, RE-oleate precursors were prepared using GdCl_3_·6H_2_O (0.25 mmol), YCl_3_·6H_2_O (0.55 mmol), YbCl_3_·6H_2_O (0.18 mmol), ErCl_3_·6H_2_O (0.02 mmol), and sodium oleate (3.1 mmol). The synthetic process was the same as that for the synthesis of the blue-emitting core UCNPs.

The RECl_3_·6H_2_O (RE = Y (0.55 mmol), Nd (0.4 mmol), and Yb (0.05 mmol)) precursors were used to synthesize the Li(Gd,Y)F_4_:Yb,Er@LiYF_4_:Nd,Yb C@S UCNPs. Other synthetic procedures were identical to those for the Li(Gd,Y)F_4_:Yb,Tm@LiYF_4_:Nd,Yb C@S UCNPs.

The red-emitting NaGdF_4_:Yb,Ho,Ce core UCNPs were synthesized by slightly modifying the method that was described in our previous paper [1]. The GdCl_3_·6H_2_O (0.5 mmol), YbCl_3_·6H_2_O (0.18 mmol), HoCl_3_·6H_2_O (0.02 mmol), CeCl_3_·7H_2_O (0.3 mmol), and sodium oleate (3.1 mmol) were mixed with DIW (3 mL), ethanol (3.5 mL), and hexane (7 mL), and the mixture was reacted at 70 °C for 4 h to prepare RE-oleate precursors. The RE-oleate, OA (6 mL), and ODE (15 mL) were mixed and the mixture was reacted at 150 °C for 40 min. The 10 mL of MeOH that contained NaOH (2.5 mmol) and NH_4_F (4 mmol) was added to the reaction solution. The reaction solution was heat-treated at 300 °C for 90 min under Ar atmosphere. The synthesized core UCNPs were washed with ethanol and hexane and then dispersed in 10 mL of hexane.

To synthesize NaGdF_4_:Yb,Ho,Ce@NaYF_4_:Nd,Yb C@S UCNPs, the mixed solution of RE-oleate (RE = Y (0.45 mmol), Nd (0.5 mmol), Yb (0.05 mmol)), OA (6 mL), and ODE (15 mL) was heat-treated at 150 °C. After cooling the mixed solution, the core solution was added to the reaction flask and the MeOH solution containing NaOH (2.5 mmol) and NH_4_F (4 mmol) was injected into the mixed solution. The mixed solution was reacted at 300 °C for 110 minutes under Ar atmosphere. The synthesized C@S UCNPs were washed and dispersed in 10 mL of hexane.

To obtain dye-sensitized C@S UCNPs, the surface modification of C@S UCNPs was performed by slightly modifying the method reported by Murray’s group [27]. After adding 1 mL of hexane to 1 mL C@S solution, 2 mL of acetonitrile and NOBF_4_ were added, followed by shaking. Then 4 mL of toluene were added to the mixed solution to precipitate the BF_4_^−^-modified C@S UCNPs and the precipitates were separated by centrifugation. Finally, BF_4_^−^-modified C@S UCNPs were dispersed in 20 mL of dimethylformamide (DMF).

The BF_4_^−^-modified C@S UCNPs and various concentrations of IR-808 dyes were mixed and reacted for 2 h under Ar atmosphere. After the reaction was completed, the dye-sensitized C@S UCNPs were dispersed in DMF.

The absorption spectra of the IR-808 dye, core UCNPs, C@S UCNPs, and dye-sensitized C@S UCNPs were obtained using a PerkinElmer (Waltham, MA, USA) Lambda 25 UV/vis spectrophotometer (scan speed = 480 nm min^−1^). The photoluminescence (PL) spectra were recorded by a PL/PLE500 device (PSI Trading Co., Ltd., Gyeonggi-do, Korea) with an 800 nm NIR light-emitting continuous-wave (CW) laser (CNI Co., Changchun, China). The transmission electron microscopy (TEM) images of core and C@S UCNPs were obtained using a Tecnai F20 G^2^ transmission electron microscope (FEI Co., Hillsboro, OR, USA) at 200 kV. A Bruker (Billerica, MA, USA) D8 ADVANCE diffractometer using Cu Kα radiation was used for the X-ray diffraction (XRD) characterization. 

## 3. Results and Discussion 

### 3.1. Characterization of the IR-808 Dye

**Figure 1:**
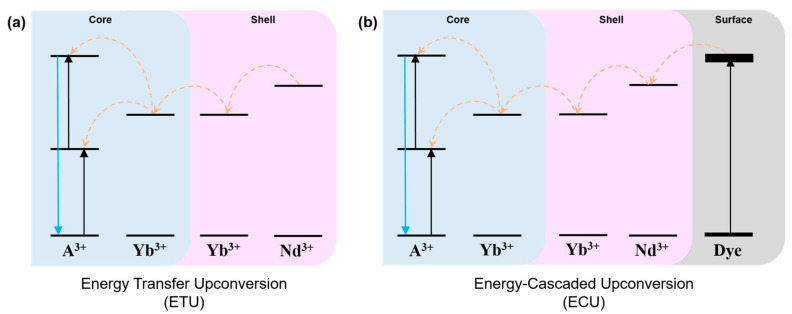
Schematic illustration showing the upconversion luminescence (UCL) through (**a**) the energy transfer upconversion (ETU) process via energy migration and (**b**) the energy-cascaded upconversion (ECU) process via organic dye sensitizer.

The lanthanide-doped UCNPs emit visible light through the energy transfer upconversion (ETU) process [28]. The UCNPs consist of sensitizer ions that absorb external energy and activator ions that emit visible light through the energy transfer from the sensitizers (Figure 1a) [29]. In contrast, dye-sensitized UCNPs emit visible light through the energy-cascaded upconversion (ECU) process [22]. The ECU process was carried out by dyes and three types of lanthanide ions (sensitizer, accumulator, and activator) in the core and C@S UCNPs, and the ECU process for dye-sensitized UCNPs is shown in Figure 1b. The dye conjugated to the surface of the UCNPs efficiently absorbs external energy and transfers the absorbed energy to the sensitizers of the UCNPs. Then, the energy is transferred to the accumulators in the core and shell, and finally to the activators in the core followed by UCL from the activators [22,30,31].

**Figure 2:**
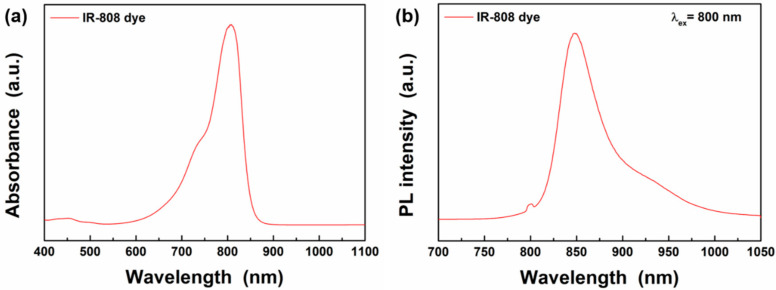
(**a**) Absorption and (**b**) photoluminescence (PL) spectra of IR-808 dyes under 800 nm excitation.

In this study, we synthesized the IR-808 dye by adapting the method reported by Prasad’s group to enhance UCL from the C@S UCNPs [22]. The synthetic method of IR-808 dye is described in Supplementary Materials and schematic illustration for the synthesis of the IR-808 dye is shown in Scheme S1 in Supplementary Materials. The synthesized IR-808 dye was confirmed by nuclear magnetic resonance (NMR) spectroscopy (Figure S1, Supplementary Materials). Figure 2 exhibits the absorption and PL spectra of the IR-808 dye. It was confirmed that the IR-808 dye showed maximum absorbance at 808 nm and a wide absorption band ranging from 650 nm to 850 nm, and it showed a broad emission band at around an 800 nm–950 nm range under 800 nm NIR light. Specifically, the emission spectrum of the IR-808 dye is well matched with the absorption wavelengths (745, 800, and 860 nm) of Nd^3+^ ions [11]. Therefore, in order to efficiently transfer the energy absorbed by the dye to the UCNPs, we synthesized C@S UCNPs where Nd^3+^ ions were doped into the shell as the sensitizers and Yb^3+^ ions were doped into the core and shell as the accumulators for the energy transfer to the activators in the core.

### 3.2. Characterization of Blue-, Green-, and Red-Emitting UCNPs

**Figure 3:**
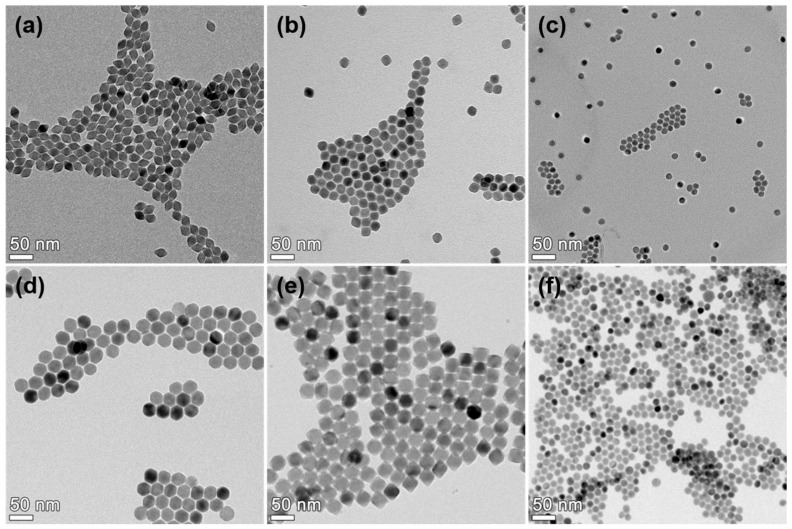
Transmission electron microscopy (TEM) images of (**a**) Li(Gd,Y)F_4_:Yb,Tm core upconversion nanophosphors (UCNPs), (**b**) Li(Gd,Y)F_4_:Yb,Er core UCNPs, (**c**) NaGdF_4_:Yb,Ho,Ce core UCNPs (**d**) Li(Gd,Y)F_4_:Yb,Tm@LiYF_4_:Nd,Yb C@S UCNPs, (**e**) Li(Gd,Y)F_4_:Yb,Er@LiYF_4_:Nd,Yb C@S UCNPs, and (**f**) NaGdF_4_:Yb,Ho,Ce@NaGdF_4_:Nd,Yb C@S UCNPs.

Figure 3 shows the TEM images of blue-emitting Li(Gd,Y)F_4_:Yb,Tm, green-emitting Li(Gd,Y)F_4_:Yb,Er, and red-emitting NaGdF_4_:Yb,Ho,Ce core UCNPs. In the TEM images, a diamond-like shape is observed (Figure 3a,b). However, polyhedral morphologies were observed in the scanning electron microscopy (SEM) images, as shown in Figure S2. In our previous study, the Li(Gd,Y)F_4_ host crystal has a tetragonal structure and it exhibits a tetragonal bipyramidal morphology to minimize surface energy by exposing the {101} planes [32]. As shown in high-resolution TEM images, the {101} planes of the Li(Gd,Y)F_4_:Yb,Tm and Li(Gd,Y)F_4_:Yb,Er UCNPs were exposed to the surface (Figure S3). Thus, the TEM images combined with the SEM images indicate that the blue- and green-emitting core UCNPs showed tetragonal bipyramidal morphologies. The sizes of blue- and green-emitting core UCNPs were measured to be 18.9 nm ± 1.0 nm × 20.7 nm ± 1.3 nm (average short edge ± standard deviation × average long edge ± standard deviation) and 21.1 nm ± 1.1 nm × 21.8 nm ± 1.4 nm, respectively. The Nd^3+^ ion-doped shells were grown on the cores to enhance the UCL by reducing the surface defect sites and efficiently transferring the external energy from the IR-808 dye to the cores [22]. Figure 3d,e shows TEM images of blue-emitting Li(Gd,Y)F_4_:Yb,Tm@LiYF_4_:Nd,Yb C@S UCNPs and green-emitting Li(Gd,Y)F_4_:Yb,Er@LiYF_4_:Nd,Yb C@S UCNPs. The sizes of blue- and green-emitting C@S UCNPs were measured to be 29.8 nm ± 1.3 nm × 29.9 nm ± 1.4 nm and 34.0 nm ± 1.6 nm × 34.0 nm ± 1.3 nm, respectively. For both C@S UCNPs, the shell thicknesses were 5.0 nm and 6.3 nm, respectively. In contrast, red-emitting core UCNPs exhibited a spherical shape with a diameter of 12.6 nm ± 1.2 nm, and the red-emitting C@S UCNPs also showed a spherical shape with larger size (15.7 ± 1.2 nm), as displayed in the TEM images of Figure 3c,f. Consequently, the TEM analysis showed that the synthesized core and C@S UCNPs were monodispersed.

The XRD patterns of the blue-, green-, and red-emitting C@S UCNPs are shown in Figure S3. From the XRD results, it was confirmed that the blue- and green-emitting C@S UCNPs have a single tetragonal phase and the red-emitting C@S UCNPs have a single hexagonal phase. It is noted that no impurity phases were formed during the syntheses of the C@S UCNPs. 

**Figure 4:**
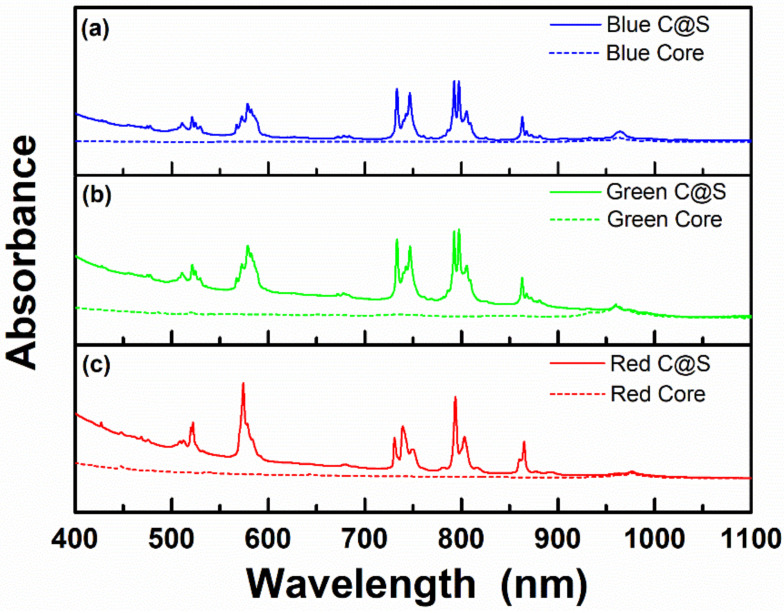
Absorption spectra of (**a**) Li(Gd,Y)F_4_:Yb,Tm core UCNPs (blue dotted line), Li(Gd,Y)F_4_:Yb,Tm@LiYF_4_:Nd,Yb C@S UCNPs (blue solid line), (**b**) Li(Gd,Y)F_4_:Yb,Er core UCNPs (green dotted line), Li(Gd,Y)F_4_:Yb,Er@LiYF_4_:Nd,Yb C@S UCNPs (green solid line), and (**c**) NaGdF_4_:Yb,Ho,Ce core UCNPs (red dotted line), NaGdF_4_:Yb,Ho,Ce@NaGdF_4_:Nd,Yb C@S UCNPs (red solid line).

Figure 4 shows the absorption spectra of the synthesized core and C@S UCNPs. As shown in Figure 4, the blue- and green-emitting UCNPs with a tetragonal bipyramidal morphology showed similar absorption properties to the red-emitting UCNPs with a spherical shape. In the blue-, green-, and red-emitting cores, there were no absorption peaks in the 800 nm region. In contrast, for C@S UCNPs, the absorption peaks were observed in the region of 700 nm to 850 nm due to ^4^I_9/2_ → ^4^F_7/2_, ^4^I_9/2_ → ^4^F_5/2_, and ^4^I_9/2_ → ^4^F_3/2_ transitions of Nd^3+^ ions [11,33]. As mentioned above, due to these absorption peaks of Nd^3+^ ions, external energy absorbed by IR-808 dyes can be effectively transferred to the C@S UCNPs [22].

### 3.3. IR-808 Dye-Sensitized C@S UCNPs

The as-synthesized C@S UCNPs were coated with OA ligand, and surface modification of UCNPs was required for conjugation with the IR-808 dye. In this study, OA-capped C@S UCNPs (OA-C@S UCNPs) were modified with BF_4_^−^ and the surface of the BF_4_^−^-modified C@S UCNPs (BF_4_^−^-C@S UCNPs) was investigated by Fourier-transform infrared (FT-IR) spectroscopy. Figure S5 shows the FT-IR spectra of the OA-C@S UCNPs and BF_4_^−^-C@S UCNPs. The OA-C@S UCNPs showed symmetric and asymmetric C-H stretching vibration peaks at 2852 cm^−1^ and 2922 cm^−1^, respectively [34]. In contrast, the peak due to BF_4_^−^ newly appeared at 1097 cm^−1^ for the BF_4_^−^-C@S UCNPs, and a peak was also newly observed at 1660 cm^−1^ due to the C=O stretching vibration of DMF molecules [34,35]. These results show that surface modification with BF_4_^−^ was successful [34]. When the BF_4_^−^-C@S UCNPs were conjugated with the IR-808 dye, BF_4_^−^ existing on the surface of UCNPs was exchanged with the carboxyl group of the IR-808 dye, giving rise to IR-808 dye-sensitized UCNPs [35]. The FT-IR spectra of the IR-808 dye and the IR-808 dye-sensitized UCNPs are also shown in Figure S6. The IR-808 dye showed C=C skeleton vibration of benzene peak at 1538 cm^−1^, C-H bending vibration peak at 1395 cm^−1^, and C-N stretching vibration peak at 1252 cm^−1^ [36,37]. Additionally, the IR-808 dye-sensitized UCNPs exhibited FT-IR peaks at 1542 cm^−1^, 1399 cm^−1^, and 1256 cm^−1^, respectively. These results indicate that the IR-808 dye was successfully conjugated with the C@S UCNPs.

**Figure 5:**
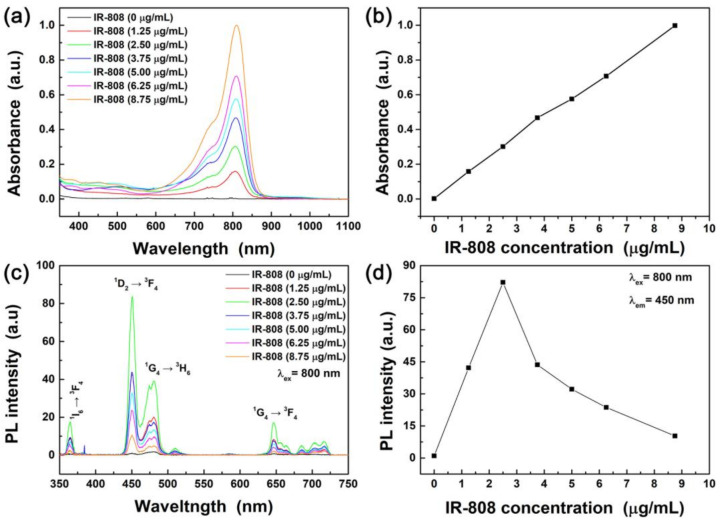
(**a**) Absorption spectra of IR-808 dye-sensitized Li(Gd,Y)F_4_:Yb,Tm@LiYF_4_:Nd,Yb C@S UCNPs. (**b**) Absorbance at 808 nm as a function of IR-808 concentrations. (**c**) PL spectra of IR-808 dye-sensitized Li(Gd,Y)F_4_:Yb,Tm@LiYF_4_:Nd,Yb C@S UCNPs under 800 nm NIR excitation. (**d**) Maximum PL intensity of IR-808 dye-sensitized Li(Gd,Y)F_4_:Yb,Tm@LiYF_4_:Nd,Yb C@S UCNPs as a function of IR-808 dye concentration.

Figure 5 shows the absorption and PL spectra of the IR-808 dye-sensitized blue-emitting C@S UCNPs. It was confirmed that the absorbance of the IR-808 dye-sensitized C@S UCNPs increased as the concentration of the IR-808 dye conjugated to the C@S UCNPs increased (Figure 5a). Figure 5b shows the absorbance of the IR-808 dye-sensitized C@S UCNPs at 808 nm with varying concentrations of the IR-808 dye, confirming that the absorbance was linearly increased with the concentration of the IR-808 dye. As shown in Figure 5c, sharp emission peaks were observed due to the ^1^I_6_ → ^3^F_4_, ^1^D_2_ → ^3^F_4_, ^1^G_4_ → ^3^H_6_, and ^1^G_4_ → ^3^F_4_ transitions of Tm^3+^ ions under 800 nm excitation [38]. To optimize UCL intensity, the concentration of the IR-808 dyes was varied and the strongest PL intensity was observed at a concentration of 2.5 μg mL^−1^. The PL intensity of the IR-808 dye-sensitized C@S UCNPs was enhanced by 87-fold compared with OA-C@S UCNPs.

**Figure 6:**
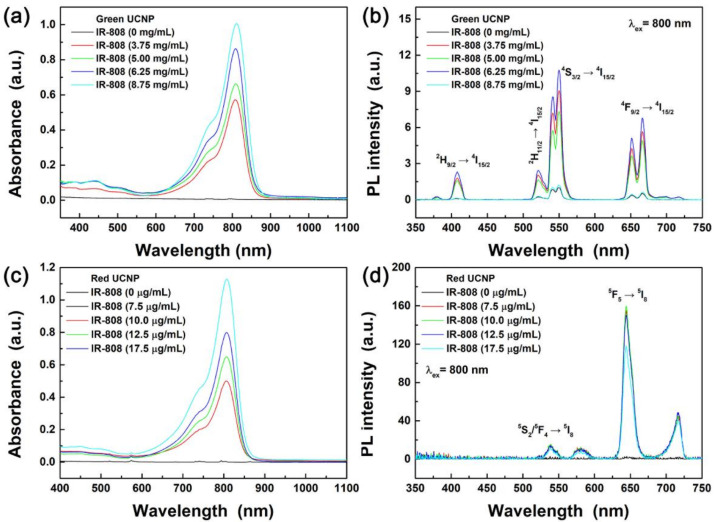
(**a**) Absorption and (**b**) PL spectra of IR-808 dye-sensitized Li(Gd,Y)F_4_:Yb,Er@LiYF_4_:Nd,Yb C@S UCNPs. (**c**) Absorption and (**d**) PL spectra of IR-808 sensitized NaGdF_4_:Yb,Ho,Ce@NaGdF_4_:Nd,Yb C@S UCNPs under 800 nm NIR light.

The absorption and PL spectra of the IR-808 dye-sensitized green- and red-emitting C@S UCNPs are exhibited in Figure 6. The higher the concentration of the IR-808 dye in the dye-sensitized UCNPs, the higher the absorbance of the IR-808 dye-sensitized C@S UCNPs (Figure 6a). The IR-808 dye-sensitized green-emitting C@S UCNPs showed sharp peaks peaking at 408, 521, 550, and 666 nm due to ^2^H_9/2_ → ^4^I_15/2_, ^2^H_11/2_ → ^4^I_15/2_, ^4^S_3/2_ → ^4^I_15/2_, and ^4^F_9/2_ → ^4^I_15/2_ electronic transitions of Er^3+^ ions under 800 nm excitation (Figure 6b) [39]. When the 6.25 μg mL^−1^ dye was conjugated to the green-emitting C@S UCNPs, the green UCL was enhanced by 10.8-fold compared with the green-emitting OA-C@S UCNPs without dye conjugation. As shown in Figure 6c, absorbance of the IR-808 dye-sensitized C@S UCNPs was increased with increasing the concentration of the IR-808 dyes. Figure 6d shows Ho^3+^ characteristic peaks due to the electronic transitions from ^5^S_2_/^5^F_4_ and ^5^F_5_ levels to the ^5^I_8_ level under 800 nm excitation [1]. When the 10.0 μg mL^−1^ IR-808 dye was conjugated to the red-emitting C@S UCNPs, the red UCL was increased by 110-fold compared with the red-emitting OA-C@S UCNPs without dye conjugation.

## 4. Conclusions

We synthesized LiREF_4_^−^based blue- and green-emitting C@S UCNPs, and NaGdF_4_^−^based red-emitting C@S UCNPs, where Nd^3+^ ions were doped in the shell. After surface modification of the C@S UCNPs with BF_4_^−^, the C@S UCNPs were successfully conjugated with IR-808 dyes and they were confirmed by FT-IR analysis. The IR-808 dye-sensitized blue-emitting Li(Gd,Y)F_4_:Yb,Tm@LiYF_4_:Nd,Yb C@S UCNPs showed 87-fold UCL enhancement and the IR-808 dye-sensitized green-emitting Li(Gd,Y)F_4_:Yb,Tm@LiYF_4_:Nd,Yb C@S UCNPs showed 10.8-fold UCL enhancement compared to the blue- and green-emitting C@S counterparts. For the first time in this study, it was shown that the UCL intensities of non-NaYF_4_-based UCNPs can be significantly enhanced and the IR-808 dye-sensitized C@S UCNPs showed bright blue and green light under 800 nm excitation. In addition, IR-808 dye-sensitized red-emitting NaGdF_4_:Yb,Ho,Ce@NaYF_4_:Nd,Yb C@S UCNPs showed strong red UCL under 800 nm excitation and they exhibited 110-fold UCL enhancement compared with NaGdF_4_:Yb,Ho,Ce@NaYF_4_:Nd,Yb C@S UCNPs. This means that red UCL can also be significantly enhanced by IR-808 dye conjugation like the blue and green UCL. Since strong UCL from the IR-808 dye-sensitized C@S UCNPs under 800 nm NIR light excitation can minimize the heating effect on cells and tissues [11], they are beneficial for the application to various fields such as bio-imaging and therapy, among others.

## Supplementary Materials

The following are available online at https://www.mdpi.com/1996-1944/13/23/5338/s1, The synthesis method of IR-808 dye is described. Scheme S1: Schematic illustration showing the synthesis of the IR-808 dye; Figure S1: NMR spectrum of the IR-808 dye; Figure S2: SEM images of the blue-, and green-emitting core and C@S UCNPs; Figure S3: High resolution TEM images of the blue- and green-emitting core UCNPs; Figure S4: XRD patterns of the blue-, green-, and red-emitting C@S UCNPs; Figure S5: FT-IR spectra of the OA-C@S UCNPs and BF_4_^−^-C@S UCNPs; Figure S6: FT-IR spectra of the IR-808 dye-conjugated C@S UCNPs and IR-808 dye.
