# Supplementary material for: Bright Blue, Green, and Red Luminescence from Dye-Sensitized Core@Shell Upconversion Nanophosphors under 800 nm Near-Infrared Light
Source: Materials (Basel). 2020 Nov 25;13(23):5338. doi: 10.3390/ma13235338 (PMC7728324; doi:10.3390/ma13235338)
Supplement: Supplementary file 1 [file materials-13-05338-s001.pdf]

materials

Supplementary Materials

Bright Blue, Green, and Red Luminescence from Dye-sensitized Core@Shell Upconversion Nanophosphors under 800 nm Near Infrared Light

A-Ra Hong [1], Joon Soo Han [1], Gumin Kang [2], Hyungduk Ko [2] and Ho Seong Jang [1,*]

[1] Materials Architecturing Research Center, Korea Institute of Science and Technology, 5, Hwarang-ro 14-gil, Seongbuk-gu, Seoul 02792, Korea; 024446@kist.re.kr (A.-R.H.); jshan@kist.re.kr (J.S.H.)
[2] Nanophotonics Research Center, Korea Institute of Science and Technology, 5, Hwarang-ro 14-gil, Seongbuk-gu, Seoul 02792, Korea; guminkang@kist.re.kr (G.K.); kohd94@kist.re.kr (H.K.)
* Correspondence: msekorea@kist.re.kr; Tel.: +82-2-958-5263

Synthesis of IR-808 dye

The IR-808 dye was synthesized by adapting the method which was previously reported by Prasad's group, and the reaction process was shown in the Scheme 1 [S1]. First, 0.134 mmol of IR-783 and 0.520 mmol of 4-mercaptobenzoic acid were added to 5 ml of DMF under nitrogen atmosphere, followed by reaction for 24 h. After the completion of the reaction, dichloromethane was added to the reaction solution to precipitate the IR-808. The precipitated dark green powder was collected by centrifugation, and the obtained power was washed with ether and dried under vacuum and stored in dark condition.

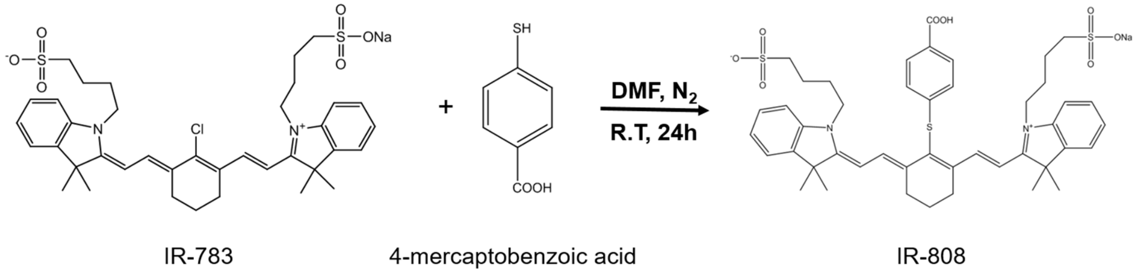

Scheme S1. Schematic illustration showing the synthesis of IR-808 dye.

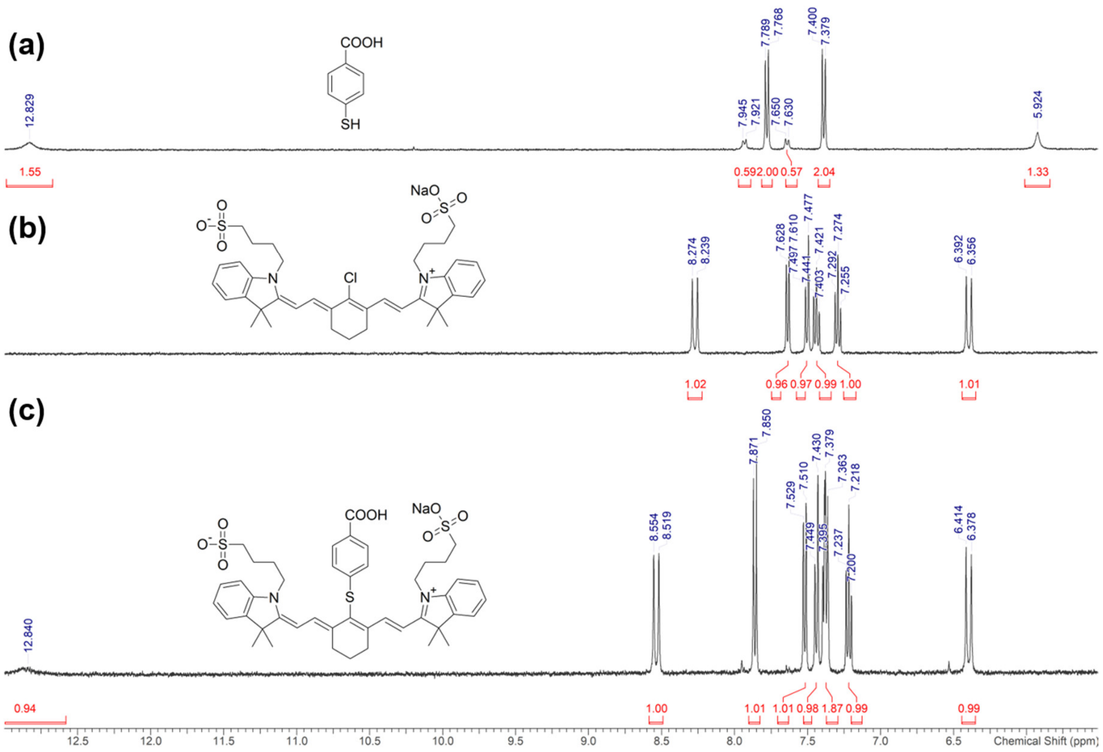

Figure S1. Partial ^{1}H NMR spectra (400 MHz, DMSO-d6) of (**a**) 4-mercaptobenzoic acid (**b**) IR-783 and (**c**) synthesized IR-808. At 7~8.5 ppm, the peaks were observed due to the benzene ring in the 4-mercaptobenzoic acid and IR-783. All peaks observed in the ^{1}H NMR spectra of the precursors were also observed in the ^{1}H NMR spectrum of the synthesized IR-808 dye. Since the electronic environment in the IR-808 dye is different from those in the 4-mercaptobenzoic acid and IR-783 due to structural changes of the compound, the positions of the aromatic peaks of the precursor were shifted from 8.25 ppm to 8.55 ppm. In the ^{1}H NMR spectrum of the IR-808 spectrum, the two doublets at 6.40 and 8.54 ppm (J = 14 Hz) can be assigned to alkenyl protons, and the significant downfield shift of the 8.54 ppm doublet indicates a change in the electronic environment due to the introduction of the mercaptobenzoic acid [S2].

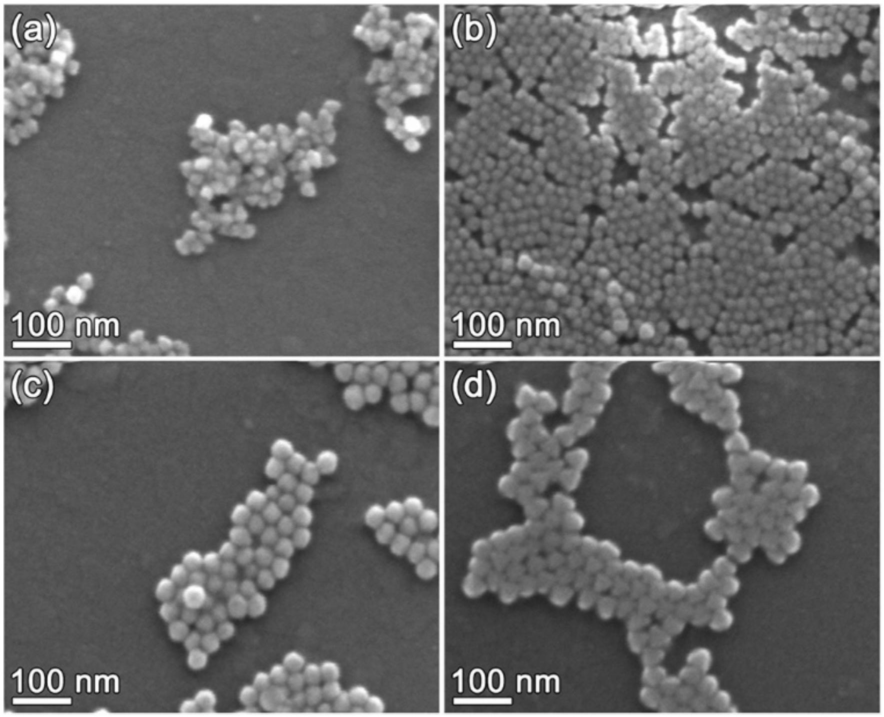

Figure S2. Scanning electron microscopy images of (**a**) Li(Gd,Y)F_4:Yb,Tm core, (**b**) Li(Gd,Y)F_4:Yb,Er core, (**c**) Li(Gd,Y)F_4:Yb,Tm@LiYF_4:Nd,Yb C@S, and (**d**) Li(Gd,Y)F_4:Yb,Er@LiYF_4:Nd,Yb C@S UCNPs.

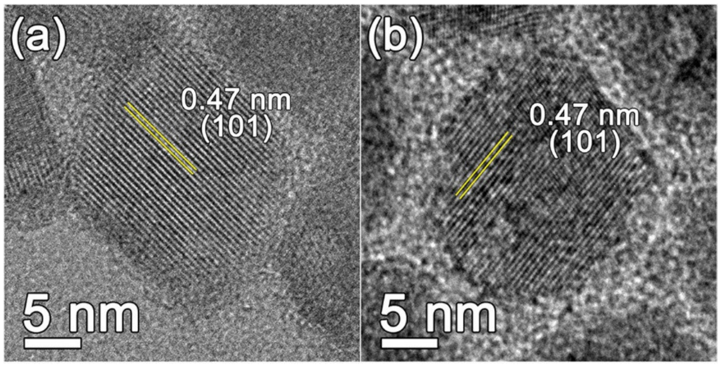

Figure S3. High resolution TEM images of (**a**) Li(Gd,Y)F_4:Yb,Tm and (**b**) Li(Gd,Y)F_4:Yb,Er UCNPs.

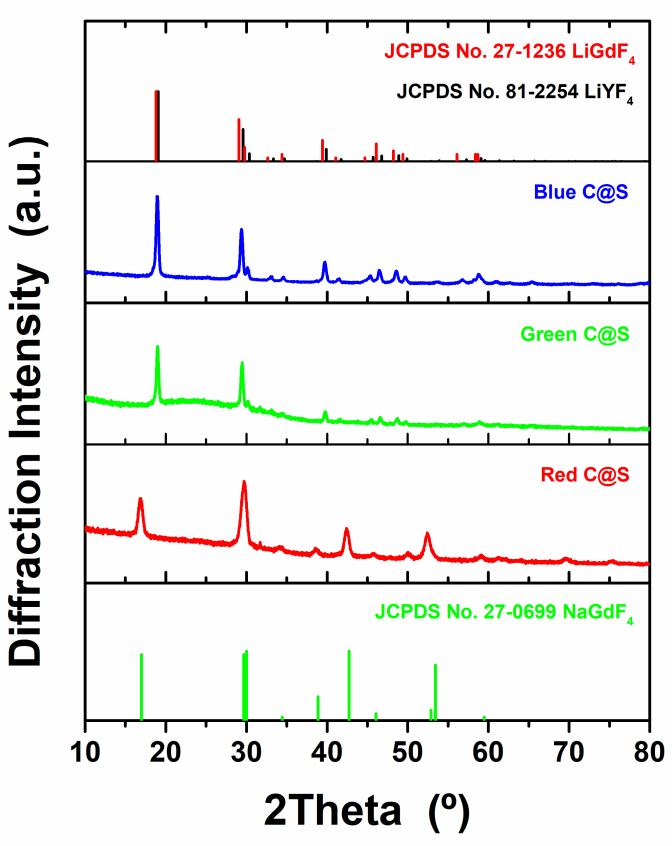

Figure S4. XRD patterns of blue-, green-, and red-emitting C@S UCNPs. The $LiYF_4$ (JCPDS No.81-2254), $LiGdF_4$ (JCPDS No. 27-1236), and β-$NaGdF_4$ (JCPDS No. 27-0699) are shown.

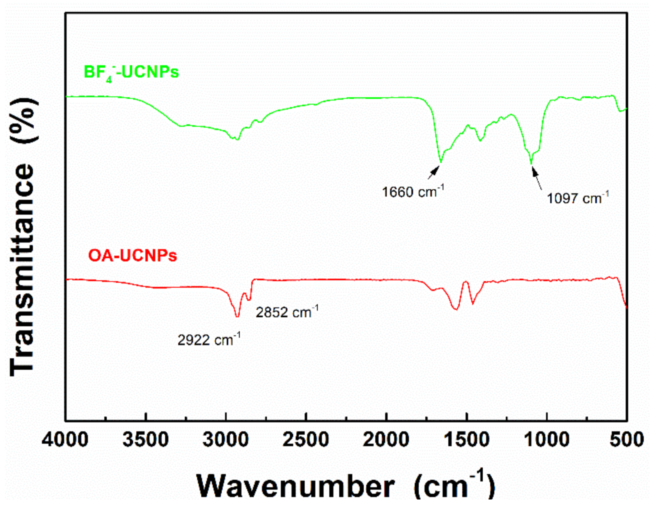

Figure S5. FT-IR spectra of OA-C@S UCNPs (red line) and BF_4^--C@S UCNPs (green line).

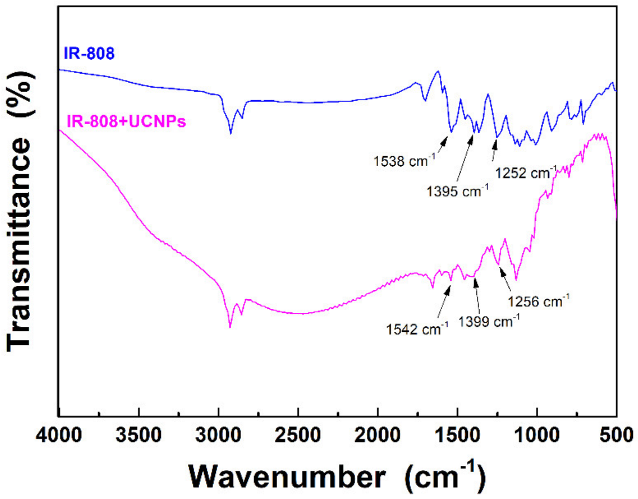

Figure S6. FT-IR spectra of IR-808 dye-conjugated C@S UCNPs (magenta line) and IR-808 dye (blue line).

References

[S1] Chen, G.; Damasco, J.; Qiu, H.; Shao, W.; Ohulchanskyy, T. Y.; Valiev, R. R.; Wu, X.; Han, G.; Wang, Y.; Yang, C.; Ågren, H.; Prasad, P. N., Energy-Cascaded Upconversion in an Organic Dye-Sensitized Core/Shell Fluoride Nanocrystal. *Nano Lett,* **2015,** *15*, 7400–7407.

[S2] Xu, J.; Sun, M.; Kuang, Y.; Bi, H.; Liu, B.; Yang, D.; Lv, R.; Gai, S.; He, F.; Yang, P., Markedly enhanced up-conversion luminescence by combining IR-808 dye sensitization and core-shell-shell structures. *Dalton Trans.* **2017,** *46*, 1495–1501.
